# Evaluation of kinetic stability and anti-staphylococcal activity of recombinant LasA protein produced in *Escherichia coli*

**DOI:** 10.22038/ijbms.2021.54563.12250

**Published:** 2021-06

**Authors:** Behnaz Rahmani, Akram Astani, Hossein Zarei Jaliani, Mohammad Hassan Kheirandish, Ahmad Mosaddegh

**Affiliations:** 1 Department of Microbiology, School of Medicine, Shahid Sadoughi University of Medical Sciences, Yazd, Iran; 2 Department of Medical Biotechnology, School of Medicine, Shahid Sadoughi University of Medical Sciences, Yazd, Iran; 3 Zoonotic Diseases Research Center, Department of Food Hygiene and Safety, School of Public Health, Shahid Sadoughi University of Medical Sciences, Yazd, Iran

**Keywords:** Antibiotic, LasA protease, MRSA, Stability, Staphylolysin

## Abstract

**Objective(s):**

*Staphylococcus aureus* has become a major clinical concern due to the growing prevalence of multi-drug resistant (MDR) strains. Enzybioticts are peptidoglycan hydrolases that are recently introduced as an alternative agent to confront the MDR strains with a more effective mechanism than conventional antibiotics. In this regard, our study aimed to evaluate the kinetic stability of LasA protease as a potent enzybiotic in the specific destruction of the *S. aureus *cell wall.

**Materials and Methods:**

The catalytic domain of the Codon-optimized LasA gene was sub-cloned into pET28a vector, and BL21 DE3 cells were used for protein expression. Recombinant LasA protein was affinity purified by Ni-NTA column and staphylolytic activity of the LasA protein against methicillin-resistant strains was evaluated by disk diffusion and MIC test. The kinetic stability was evaluated in different temperatures during 48 hr.

**Results:**

Our results revealed that LasA protein can completely prevent the growth of Methicillin-resistant *S. aureus *(MRSA) strain and inhibit the examined strain at the amount of 4 µg. furthermore, the catalytic domain of LasA protein can tolerate higher temperatures as well.

**Conclusion:**

With regard to the failure of conventional antibiotics in treatment of MRSA infections, novel agents to combat multidrug-resistant strains are needed. The present study shows that LasA protein can eradicate MRSA strains, so it can be promising for the treatment of antibiotic-resistant staphylococci infection. The kinetic stability of LasA has also confirmed the possibility of industrial-scale manufacturing for the subsequent use of the enzyme clinically.

## Introduction

Although *Staphylococcus aureus* is considered a part of the normal flora in the human body, it causes numerous bacterial infections in humans including pneumonia, bacteremia, osteomyelitis, toxic shock syndrome, and endocarditis. Generally, *S. aureus *is the agent of hospital-associated (HA) and community-associated (CA) bacterial infections in humans (1). Antibiotic resistance in *S. aureus *has occurred through horizontal gene transfer for the acquisition of mobile genetic elements (2, 3) and somatic mutations to alter drug binding sites in the bacteria (4). After observation of resistance to penicillin in *S. aureus *species, methicillin as a semisynthetic penicillin derivative was designed to fight resistance (5). The problem still exists and is increasing, since methicillin-resistant *S. aureus* (MRSA) has raised serious problems for treatment of infections and it can acquire resistance to various drugs and antibiotics(1). So, *S. aureus *is one of the most threatening microorganisms and its antibiotic resistance has spread out among *S. aureus *species (6).

By now it is necessary for *S. aureus *and also for a couple of other resistant infectious species to find alternative routine antibiotics (7). Despite recognition of microorganism-degrading enzymes from the last century, they have not yet been extensively applied and routine antibiotics are used in nearly all the clinical treatments (8). Enzybiotics are composed of lytic enzymes that are found naturally in bacteria, phages, and body fluids. They consist of lysins, autolysins, and bacteriocins. they have peptidoglycan hydrolase function with minimal effect on commensal flora (9, 10). Enzybiotics can also prevent the formation of biofilms by eliminating bacteria from mucosal surfaces. Therefore, they can overcome sepsis, endocarditis, pneumonia, meningitis, and nasopharyngeal, skin, and vaginal decolonization. Enzybiotics usually do not have adverse effects and there is usually no resistance to them. A lower probability of the resistance of bacteria to enzybiotics than routine antibiotics can be attributable to the highly conserved and critical peptidoglycan components which are the target of enzybiotics (11).

LasA protease is one of the bacteriocins and is known as a staphylolysin, it’s produced by *Pseudomonas aeruginosa *and acts against antibiotics resistant staphylococci like MRSA effectively. This endopeptidase can cleave peptide bonds following Gly-Gly in the pentaglycine bridge that is necessary for stabilization in the cell wall of *S. aureus* (12). In the present study, the catalytic domain of the LasA protein was expressed separately as a recombinant protein in *E. coli* and its staphylolytic activity against MRSA and kinetic stability in different temperatures during 48 hr has been explored.

## Materials and Methods

***Molecular cloning***

The gene fragment encoding the catalytic domain of LasA protein after codon optimization was ordered to be chemically synthesized by Pishgaman Gene Transfer Company. The gene delivered in pUC57 plasmid was transformed into *E. coli* DH5α competent cells. The construct was extracted using the QIAprep spin miniprep kit from transformants. This construct was digested using NcoI and XhoI restriction enzymes, and the LasA gene was introduced into pET28a(+) bacterial expression vector.

LasA-pET28a expression construct was transformed into DH5α competent cells and colony-PCR was performed with universal primers T7 promoter and T7 terminator as follows; T7P sequence: GAAATTAATACGACTCACTATAG, and T7T sequence: GCTAGTTATTGCTCAGCGG. PCR program steps were as follow; an initial step of 95 °C for 5 min, (95 °C for 30 sec, 56°C for 30 sec, and 72°C for 40 sec) for 30 cycles, and a final extension of 72 °C for 10 min.

Positive clones were confirmed by DNA sequencing. LasA-pET28a construct was extracted from DH5α transformants and was transformed into competent BL21 (DE3) bacteria.

***Expression, induction, and purification***

The colonies of BL21 with LasA-pET28a construct were tested in terms of expression and the best colonies were selected for protein purification. Briefly, one colony was cultured for inoculation of a 100-ml LB medium. The culture was incubated at 120 rpm under shaking, and when the OD^600 nm ^reached 0.6, an inducer (0.2 mM IPTG) was added to the culture.

After overnight induction, bacteria were harvested by centrifugation at 5,000 rpm for 3 min.

The bacterial pellet was resuspended with lysis buffer (25 mM HEPES, pH 7.0, 600 mM NaCl, 10 mM 2ME, 10% glycerol). The bacteria were lysed with bead-beating (15 sec periods for 10 min). Ni-NTA agarose column (Qiagen Inc) was prepared for purification and the soluble fraction of lysed bacteria was loaded on a column. All buffers of the purification steps were the same as the lysis buffer except for the 300 mM of imidazole in the elution buffer. Fractions were analyzed by 15% SDS-PAGE. A protein size marker (Sinaclon cat no. PR911641) was used. The fractions containing the desired protein band were dialyzed against the storage buffer (50 mM NaCl, 25 mM HEPES buffer, and 20% glycerol).

***Disk diffusion***

A bacterial suspension of MRSA was prepared based on the 0.5 McFarland standard. The surface of the Muller-Hinton agar plate was perfectly impregnated with *S. aureus* suspension and the disks containing the purified catalytic domain of LasA protein and storage buffer (as a negative control) were placed on the plate. After overnight incubation, sensitivity to recombinant LasA protein was determined, and the appearance of the halo of lack of bacterial growth was assessed. 

***MIC test***

0.5 McFarland MRSA was cultured in Muller-Hinton broth in a 96-well plate. Serial dilutions of recombinant LasA protein were added to the bacteria. The plate also included positive control (bacteria without the LasA protein) and negative control (medium only). After overnight incubation at 37 °C, OD of wells was read and MIC concentration was determined (13).

***MBC test***

After completing the MIC test, the lowest concentration at which LasA protein would kill MRSA was determined. A sample from the dilution representing MIC and at least two of the more concentrated LasA protein dilutions were cultured on Muller-Hinton agar Medium. After 24 hr of incubation, the lowest concentration at which the bacteria would not grow was considered as MBC concentration (14).

***Enzyme kinetic stability assay***

For assessment of irreversible thermal inactivation of LasA protein, a sample of the purified LasA protein was treated at different temperatures (37, 52, and 75 °C), and the residual staphylolytic activity was measured at defined time intervals. MRSA in accordance with McFarland standards was prepared to be cultured with aliquots of the treated LasA protein at different time intervals. The culture was incubated overnight, and measured ODs were compared with control (un-treated LasA protein sample). The time required for the activity of the treated LasA protein to reach half of the initial level is defined as T_1/2 _(15).

## Results

***Cloning, expression induction, and purification***

**Figure 1:**
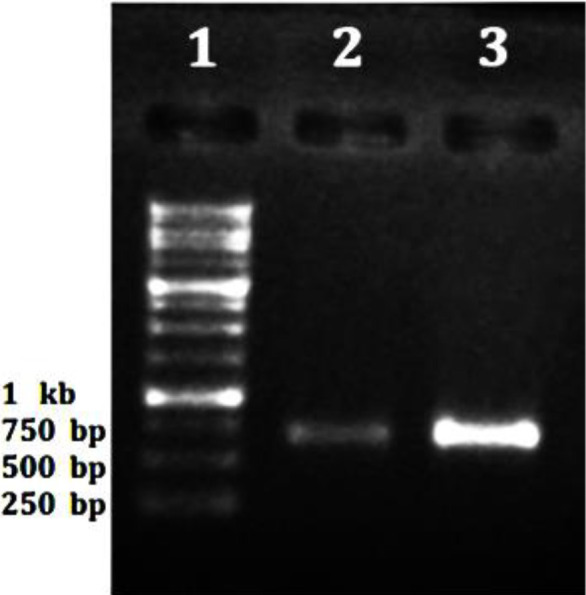
Colony-PCR of recombinant construct pET28a-LasA

**Figure 2:**
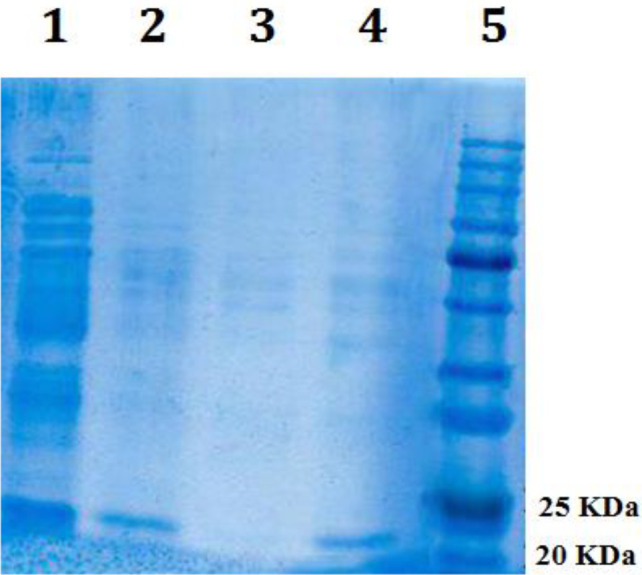
His-tagged catalytic domain of LasA protein purification

The gene fragment encoding catalytic domain of LasA protein was inserted into pET28a expression vector and recombinant construct pET28a-LasA was verified using colony-PCR (Figure 1) and sequencing. After transformation of pET28a-LasA construct into BL21 (DE3) cell, expression of recombinant LasA protein was induced by IPTG. The recombinant protein was expressed and the size of the protein was confirmed to be about 21 kDa by SDS-PAGE analysis. C-terminal his-tagged recombinant LasA protein was affinity-purified by using a mini-column filled with Ni-NTA agarose resin. The purity of the recombinant LasA protein was calculated to be about 59% (calculated by ImageJ software) as demonstrated in Figure 2 as a band on SDS-PAGE. 

***Disk diffusion***

**Figure 3:**
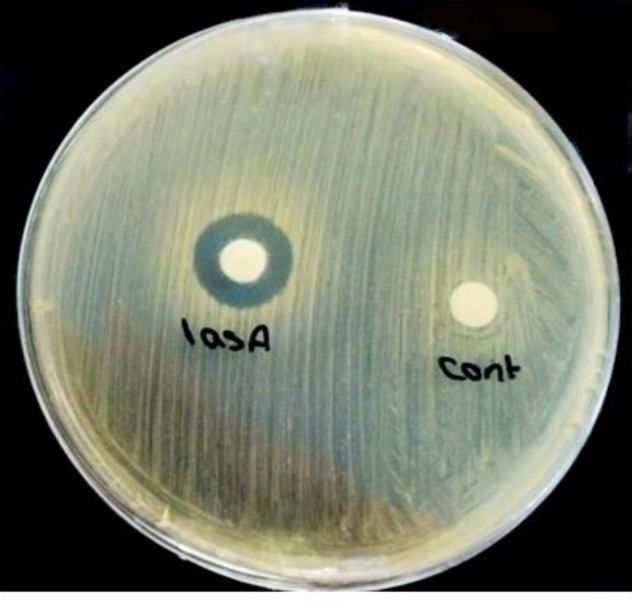
LasA protein staphylolytic activity assessment by disk diffusion

In the disk diffusion method, the halo of lack of bacterial growth around the disk contained the LasA protein; Muller-Hinton agar plate impregnated with MRSA, showed that C-terminal his-tagged recombinant LasA protein inhibited the growth of MRSA bacteria (Figure 3).

***MIC test***

**Figure 4:**
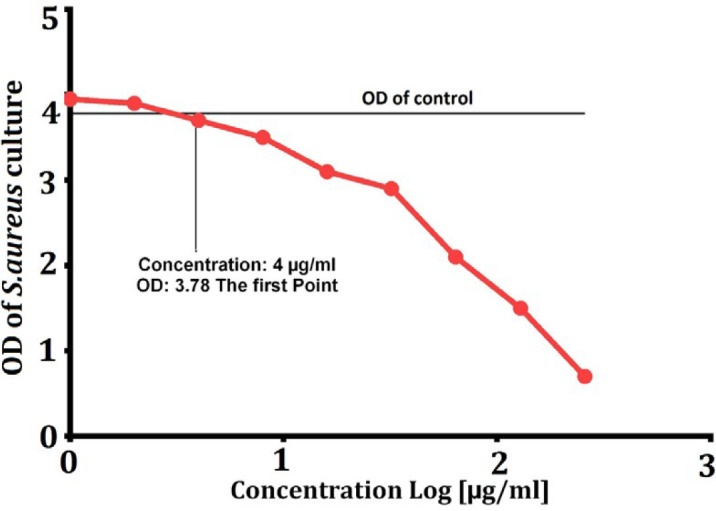
MIC test based on the minimum inhibitory concentration of LasA protein. The concentration of LasA protein at MIC test was measured to be 4 μg/ml

LasA recombinant protein at concentrations ranging from 1-258 µg/ml was evaluated for MIC testing and the MIC concentration for LasA protein was determined to be equivalent to 4 µg/ml (Figure 4).

***MBC test***

The MBC test was performed at MIC concentration and six concentrations below MIC. MBC concentration for the recombinant catalytic domain of LasA protein was evaluated and calculated to be 258 µg/ml.

***Kinetic stability***

**Figure 5:**
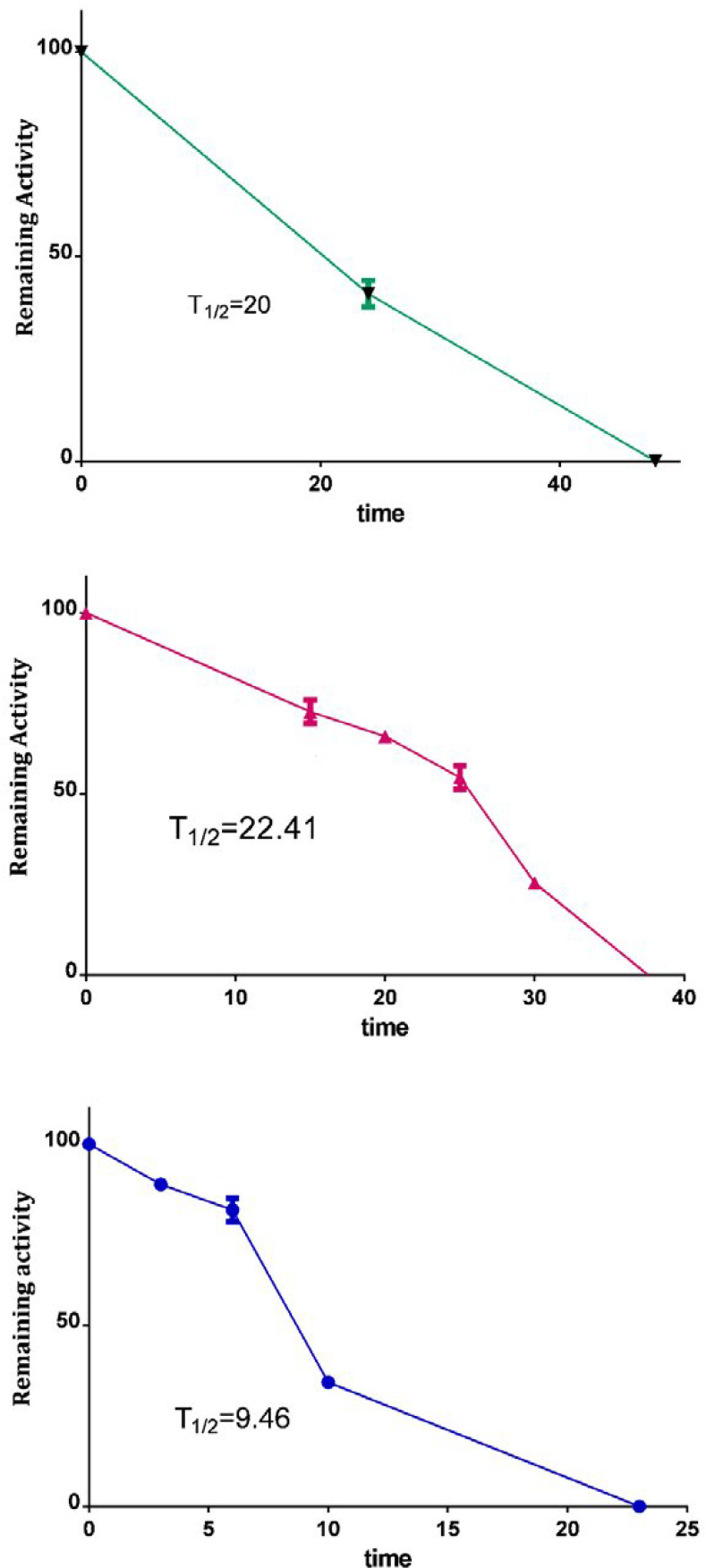
Evaluation of kinetic stability of LasA protein at defined time intervals at 3 temperatures

The times required for the residual staphylolytic activity to be 50% (T_1/2_) for the catalytic domain of LasA protein at 37 °C, 52 °C, and 75 °C were 20, 22.41, and 9.46, respectively. This domain retained more than 80% of their original activity at 37 and 52 °C after 10 hr of incubation and after 5 hr of incubation at 75 °C (Figure 5).

Colony-PCR of the recombinant construct pET28a-LasA in DH5a was performed with universal primers T7 promoter and T7 terminator. Lane 1; 1 kb ladder, lane 2 and 3; PCR products of the respected transformants

Affinity purification of the recombinant LasA protein was performed by using a mini-column filled with Ni-NTA agarose resin. Lane 1; pellet of the lysate of induced bacteria, lane 2; soup of the lysate, lane 3; first drops of the wash, lane 4; first fraction of the elution, lane 5; pre-stained protein size marker

Disk diffusion assessment tested the staphylolytic activity of purified recombinant catalytic domain of LasA protein. The zone of inhibition (clear halo) around the disks loaded with LasA catalytic domain is sharp but the control disk did not affect the growth of *Staphylococcus aureus*

Kinetic stability of the catalytic domain of LasA protein was measured at 37 °C, 52 °C, and 75 °C, and T1/2 was calculated. In Figure 5 the first diagram is related to 37 °C, the second diagram is related to 52 °C, and the third diagram is related to 75 °C

## Discussion

Emergence of bacterial resistance is quickly occurring around the world. Bacterial infection has become a global problem though decades have passed since the discovery of the first antibiotic.

Excessive and inappropriate consumption of antibiotics is the main cause of the antibiotic resistance crisis. Nowadays, about 70 % of bacterial infection agents in hospitals have become resistant to at least one antibiotic and some bacteria are resistant to all antibiotics and the only way to treat them is to use potentially toxic drugs (16, 17).

In the era of ineffectiveness of antibiotics, using enzybioticts with new and different strategies against bacteria is in progress (7).

The LasA protein has staphylolytic activity and is secreted from *P. aeruginosa*. The gene for the LasA protein was sequenced in 1988(18). This protein which is also called staphylolysin, belongs to the b-lytic endopeptidase family of proteases and hydrolyzes the glycine peptide bonds in the* S. aureus *cell wall (19). It has been shown that LasA can enhance elastolytic activity by cleaving the Gly-Gly bond in elastin. It is worth mentioning that LasA itself does not have considerable elastolytic activity and just helps with other elastase enzymes to enhance their activity (17, 20)

LasA protein has shown positive results in two animal model studies in which its staphylolytic activity was examined against keratitis in the rabbit model (21) and endophthalmitis in the rat model (22). It has shown to be more efficient even than vancomycin in animal models (21), and in neither study, were there any adverse effects in the animal models.

In previous studies, LasA protein has been extracted from *P. aeruginosa *culture supernatants and purified with ion-exchange chromatography. In the present study the recombinant mature form of LasA was produced in *E. coli* and in addition to anti-staphylococcal activity, the kinetic stability of this mature form of LasA was examined for the first time. Codon-optimized gene of LasA was cloned into pET28a(+), and after protein expression and purification with Ni-NTA column, anti-staphylococcal activity and the remaining activity after incubation in different temperatures was assessed on the protein. Despite the weak binding of the C-terminal his-tagged LasA protein, the yield of the LasA protein in this study was enough to complete the analysis of the staphylolytic activity and other assays (23).

In the disk diffusion test, LasA has a significant growth inhibitory effect on MRSA. The MIC concentration of LasA was determined to be 4 µg.ml^-1^, and the minimal bactericidal concentration (MBC) was 258 µg.ml^-1^. 

Kinetic stability analysis of the LasA protein showed that this protein can be stable and active in relatively high temperatures. A mature form of LasA protein was able to withstand even 75°C temperature for 23 hr, and it still had anti-staphylococcal activity. Thermal inactivation of the recombinant catalytic domain of LasA protein revealed that this fragment of LasA protein can tolerate higher temperatures and has reasonable kinetic stability for use in pharmaceutical formulation and industrial applications. 

LasA and other enzybiotics with relatively good kinetic stability and effectiveness could be a substitute for routine antibiotics and in the future must be used more to save humanity from the crisis of antibiotic resistance.

## Conclusion

Enzybiotics with acceptable antibacterial properties could be an alternative for routine antibiotics that have low efficiency against new resistant strains. As the results of the present study showed, LasA has suitable antimicrobial activity and might be an efficient candidate for clinical use. On the other hand, we found it stable in high temperatures for subsequent industrial manipulations. However, future studies on the pharmacokinetic and pharmacodynamic profiles of these enzybiotics, *in vivo,* are required in order to establish clinical usage. 
